# The relationship between bi-spectral index and VOTE score in evaluation of drug-induced sleep endoscopy: A systematic meta-analysis

**DOI:** 10.1097/MD.0000000000035209

**Published:** 2023-09-22

**Authors:** Özlem Öner, Mustafa Cenk Ecevit, Ali Necati Gökmen

**Affiliations:** a Faculty of Medicine, Department of Anesthesiology and Reanimation, Subdivision of Critical Care Medicine, Dokuz Eylül University, Izmir, Turkey; b Faculty of Medicine, Department of Otorihinolaryngology, Dokuz Eylül University, Izmir, Turkey.

## Abstract

**Objective:**

The aim of this study was to investigate both the presence and severity of collapse in anatomical regions defined by the VOTE score (velum, orofarinx, tongue, and epiglottis), during drug induced sleep endoscopy (DISE) in patients diagnosed with obstructive sleep apnea, based on the bi-spectral index (BIS) sedation level.

**Methods:**

In order to conduct a meta-analysis of articles examining the relationship between the VOTE score and BIS sedation level in determining the presence and severity of upper airway collapse during DISE, a literature review was performed.

**Results:**

As a result of the search made in the specified databases, a total of 1864 articles were reached. Five articles included in this review that had sufficient statistical data to be included in the meta-analysis were found. A statistically significant correlation was found between the BIS sedation level and the areas of obstruction in the VOTE score. The strongest association is at the epiglottis level, followed by the velum, oropharynx, and tongue, respectively (CC: 0.639, CC: 0.53, CC: 0.49, and CC: 0.346, *P* < .001). In the subgroup analysis of publications with BIS sedation levels in the range of 60 to 65, the distribution in the epiglottis region was heterogeneous, and it was found to be statistically significant according to the random effect model (*P* < .001). The distribution in the tongue was homogeneous, and it was found to be statistically significant according to the fixed effect model (*P* < .001). When the publications in which the BIS sedation level is in the range of 65 to 75 are examined according to the areas of obstruction; the distribution in 4 anatomical regions was homogeneous and statistically significant according to the fixed effect model (*P* < .001).

**Conclusion:**

It was found that BIS sedation levels during DISE application in obstructive sleep apnea patients were associated with obstruction of the anatomical regions of the upper airway. The strongest association was found at the epiglottis level, followed by the velum, oropharynx, and tongue, respectively. It is helpful to monitor the sedation level with BIS in order to better define the collapsed areas during DISE application. However, more studies are needed to better understand the relationship between BIS sedation values and sleep stages.

## 1. Introduction

Obstructive sleep apnea (OSA) is a sleep-related respiratory disorder with a high mortality expectation, characterized by hypoxia, hypercarbia, increased sympathetic activity, and medical complications in which the upper airway is partially or completely obstructed during sleep.^[1]^

The most common surgical treatment method traditionally applied is uvulopalatopharyngoplasty, with an average success rate of 40% in all patient groups.^[2]^ The most important reason for failure is the inadequate preoperative evaluation required to determine the area or areas of the upper airway obstruction segment.^[3]^ In order to decide on the patient selection and target segment that will benefit from the surgery, the sleep surgeon should know the exact area or areas of the upper airway obstruction segment.^[4]^

Drug-induced sleep endoscopy (DISE), which has been developed in the last 3 decades, provides information about the narrowing and obstruction patterns of the upper airway in pharmacologically induced sleep.^[5]^ DISE can be performed when considering positive airway pressure alternatives such as upper airway surgery, oral appliance therapy, positional therapy, or a combination of different treatment modalities.^[6]^ Compared to the assessments made in an awake patient, DISE can change surgical treatment techniques in approximately 50% of OSA patients.^[7]^

Anesthetic and sedative agents are effective in the collapse of anatomical regions in OSA patients.^[8]^ However, the degree of this effect has not been clarified.^[9]^ It is necessary to measure the depth of sedation during DISE to simulate sleep closest to natural sleep and to understand muscle relaxation levels.^[9]^ Although there is no standard protocol for sedation application and monitoring methods in DISE, it is possible to monitor sedation with a bi-spectral index (BIS).^[10]^ BIS evaluates the electro-encephalography values by converting them into signals between 0 and 100 and gives information about the depth of sedation and the level of consciousness.^[11]^

The VOTE (velum, orofarinx, tongue, and epiglottis) classification, developed to rank the collapse patterns observed during DISE, is a collaborative comparison of studies based on the anatomical structures that contribute to upper airway obstruction segment, qualitatively evaluating the degree of UAA narrowing and providing a scientific evaluation of DISE in different centers. It is a scoring system that enables language formation.^[12,13]^

This study aims to systematically review the international literature and make a meta-analysis with available data to evaluate the relationship between the BIS sedation level during the DISE application and the VOTE score, which evaluates the obstructed areas of upper airway in patients diagnosed with OSA by polysomnography (PSG).

## 2. Material and methods

Studies were screened using the Preferred Reporting Items for Systematic Reviews and Meta-analysis statement (PRISMA) guidelines (https://www.prisma-statement.org). Since meta-analysis studies are studies based on data analysis, ethics committee approval is not required in our country. Ethics committee approval was therefore waived.

### 2.1. Study eligibility criteria

We conducted this review based on PICO question (Patients, Intervention, Comparison, Outcome), which is “Do BIS levels have any effect on VOTE score in patients over 18 years of age who were diagnosed with OSA by PSG?”

### 2.2. Inclusion criteria

All randomized controlled studies, observational (prospective or retrospective) studies evaluating the effects of BIS levels, indicating the level of sedation, on the VOTE score when applying DISE in patients over 18 years of age with PSG-confirmed OSA were included. While scanning, English, and Turkish were determined as publication languages. No publication year restrictions were imposed.

### 2.3. Exclusion criteria

Pediatric age, animal studies, anatomical, genetic, radiological, biochemical, or histopathological studies were excluded. At the same time, studies using a scoring system other than VOTE when evaluating DISE findings, studies in which the explanation of statistical data is insufficient, studies whose publication language is not English or Turkish, and studies that do not have sufficient information to perform the meta-analysis (without the primary endpoint of DISE), and other diseases and Studies focused on surgical procedures (e.g., without DISE), systematic reviews, meta-analyses, and non-original studies (letters to the editor, posters, reviews, book chapters, etc.) were excluded.

### 2.4. Literature search

**Figure 1.**
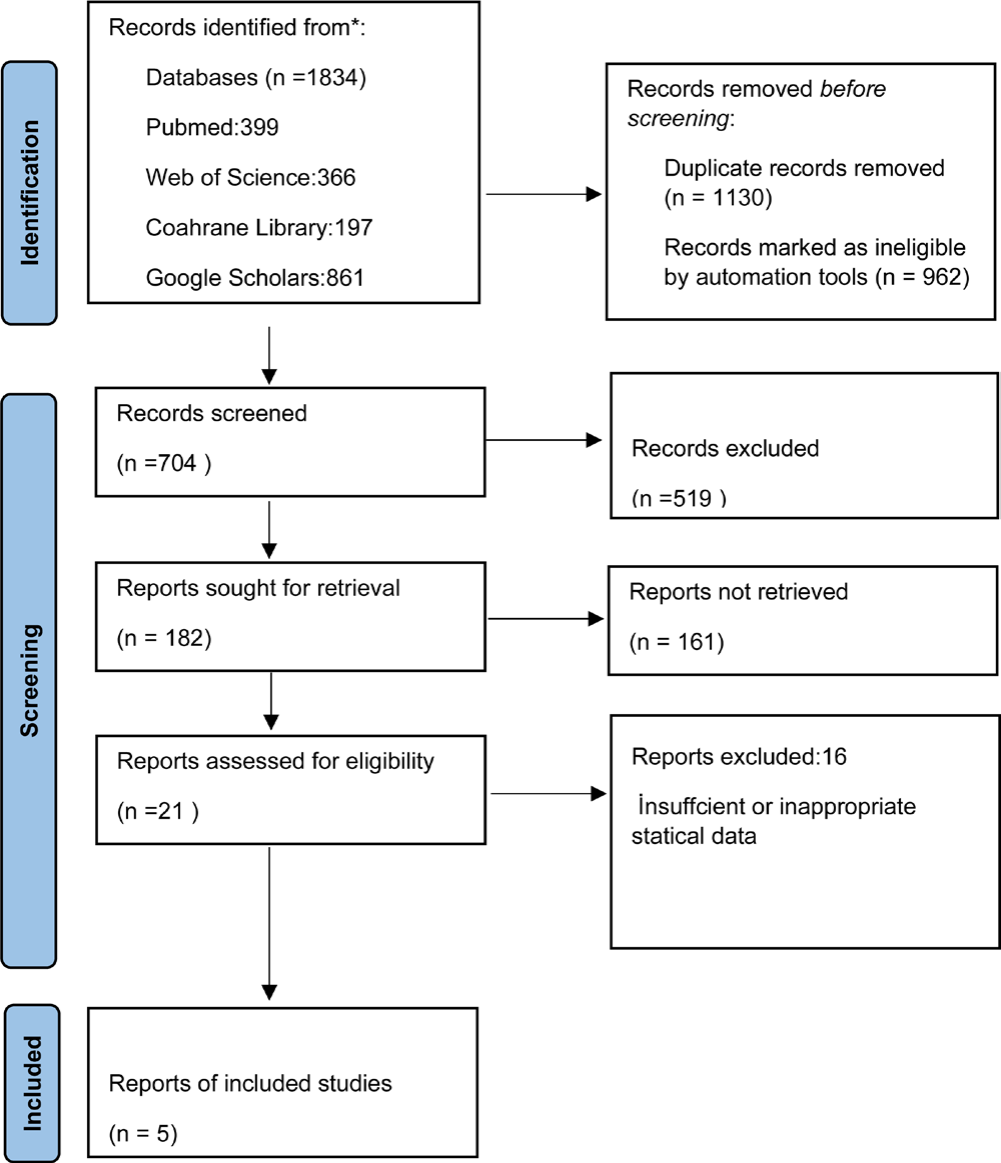
PRISMA flow chart (http://www.prisma-statement.org/).

Searches were done in the Cochrane Library, ISI Web of Knowledge (www.isiwebofknowledge.com), Pubmed (MEDLINE), Clinical Trials (ClinicalTrials.gov), and gray literature databases. Sources of gray literature included Open Gray and Google Scholar (http://scholar.google.com). In addition to the “cited by” function of Google Scholar, the “related citations” function was used to search for additional studies. Conference papers and abstracts were manually scanned in Web of Science. EndNote software (Clarivate Analytics, Philadelphia, PA) was used for duplication control. The scanning strategy following the PRISMA guidelines is shown in detail in Figure 1.

### 2.5. Search and Medical Subject Headings terms

A search began in July 2020 and was completed on March 5, 2021. Various combinations of the following Medical Subject Headings terms were used in the search strategy: “Drug-Induced Sleep Endoscopy,” “Sleep Naso-Endoscopy,” “Obstructive Sleep Apnea,” “Obstructive Sleep Apnea-Hypopnea,” “Bi-spectral Index,” ‘“BIS,” ‘‘VOTE classification,” and ‘“VOTE Scores.”

All titles and abstracts in studies were evaluated according to inclusion and exclusion criteria, included or excluded from the analysis. Full texts meeting the screening criteria were then downloaded and all reviewed.

### 2.6. Selection of studies

For eligibility assessments, the international literature was searched independently by 2 researchers. To identify which studies potentially met the criteria, the researchers identified relevant items and set inclusion and exclusion criteria. According to these criteria, a search was done in the specified databases. Differences of opinion were resolved through consensus, and if there was a lack of consensus, a solution was provided by the senior author’s final decision.

### 2.7. Primary and secondary outcomes

As the primary outcomes for the studies to be included in the meta-analysis, the grading of the collapse areas of the anatomical regions included in the VOTE score during the DISE application in patients whose diagnosis of OSA was confirmed by PSG.

In the secondary outcome, the relationship between the different values of the BIS, which shows the depth of anesthesia, and the collapsed anatomical regions evaluated according to the VOTE score are discussed.

In this meta-analysis, we investigated the relationship between BIS sedation levels and the presence and severity of collapse in 4 anatomical regions defined by the VOTE score.^[14]^ The grading categories are as follows:

Four anatomic regions: Velum, Orofarinx, Tongue, and Epiglottis.

- Grade 0: no obstruction, no vibration;
- Grade 1: partial obstruction (partial vibration);
- Grade 2: complete obstruction.

### 2.8. Data management

The spreadsheet specified in Microsoft Excel was used for data extraction. The following data were collected for each included study. These data were the names of the authors, the year of publication, the aims of the study, the country, the number of patients reported, the mean age, gender information, mean apnea-hypopnea index (AHI), BIS values, and the National Institute for Health and Clinical Excellence evaluation score calculated for each study (Table 1).

**Table 1:** Baseline characteristic of included studies.

Authors/publication years	Study	Country	Study design	Objective	Number of patients	Age, mean (SD)	Gender percent of male patients	Body mass index, mean (SD)	Apnea-Hypopnea index, mean (SD)	Bi-spectral index	Sedative agent	Main outcomes	NICE
Park et al/2019	Obstruction patterns during drug-induced sleep endoscopy vs natural sleep endoscopy in patients with obstructive sleep apnea	South Korea	Prospective cohort study	To compare the obstruction patterns during DISE with the obstruction patterns during NSE	26 patients	44.7 (10.3)	85%	26.8 (4.4)	41.9 (17.2)	65–75	Midazolam	Obstruction patterns of the upper airway appeared to be in agreement between DISE and NSE, suggesting DISE may be a reliable test	7
Park et al/2017	Effect of physical stress on drug-induced sleep endoscopy for obstructive sleep apnea	Republic of Korea	Retrospective observational study	To evaluate the impact of physical stress on DISE findings	85 patients	49.3 (15.5)	80%	25.5 (3.7)	22.2 (8.7)	60–65	Midazolam	When counseling patients on the likely value of sleep surgery based on DISE findings, stressful physical activity should be included as a contributing factor in	6
Wang et al/2018	The role of drug-induced sleep endoscopy: predicting and guiding upper airway surgery for adult OSA patients	China	Retrospective observational study	To evaluated the ability of DISE to predict the final effect of upper airway surgery and potentially to guide surgical treatment decision-making	85 patients	44.1 (10.7), n = 48 46.7 (11.2), n = 37	59%	26.2 (4.3), n = 48 27.5 (4.9), n = 37	38.3 (15.4), n = 48 36.9 (17.1), n = 37	60	Propofol	Our results suggest that DISE may help predict the final outcome of tonsillectomy, UPPPA, or a combination of the two in adult patients with OSA. The use of DISE shows potential to guide treatment decisions for individual patients with OSA	6
Won et al/2020	Correlation of site of obstruction between 2 dynamic evaluation modalities in obstructive sleep apnea patients: drug-induced sleep endoscopy and sleep videofluroscopy	Republic of Korea	Retrospective observational study	To evaluated the correlation of obstructive sites determined by DISE and sleep videoflurosscopy in OSA patients and elucidate findings that can improve the accuracy of upper airway assessment	63 patients	45.2 (18–67)	76%	25.5 (17–35.1)	29.5 (23.1)	70–80	Midazolam	They found a good overall agreement between the 2 dynamic airway evaluation modalities during drug-induced sleep, and	6
Bilal et al/2019	The evaluation of obstruction degree by position and sleep depth in sleep endoscopy	Turkey	Prospective study	Anesthesia management in OSA patients is particularly important for airway management. In our study, it was aimed to evaluate the upper airway by determining obstruction sites endoscopically according to OSA patients’ sleep depth and position	47 patients	42.2 (9.4)	80.9%	29.0 (3)	20.0 (14.9)	65–75	Propofol	Under deep sedation since obstruction is observed at all levels in the supine position, OSA in problems that may occur in the airway in the airway of lateral positioning in patients can prevent obstructions	7

DISE = drug-induced sleep endoscopy, NSE = natural sleep endoscopy, OSA = obstructive sleep apnea.

### 2.9. Quality assessment of study

The National Institute for Health and Clinical Excellence quality assessment tool evaluated the quality of the studies included in this meta-analysis.^[12]^ The quality score was calculated for each study included in this meta-analysis and is shown in Table 1.

### 2.10. Statistics

As the effect size, the contingency coefficient (CC) between the rating in the VOTE scoring system (grade 0–1–2) and the obstruction areas (velum, oropharynx, tongue, epiglottis) was used. Meta-analysis of these correlation coefficients was performed using the Fisher *Z* transform using the Hedges-Olkin (1985) method to calculate the weighted summary Correlation coefficient under the fixed effects model. CC and 95% confidence interval (CI) values are given. CC value; If it is greater than 0.5, it was evaluated as a high association, 0.3 to 0.5 moderate association, 0.1 to 0.3 low association, if less than 0.1 little if any association.^[15]^ If the correlation coefficient is; 0.10 to 0.39 was accepted as a weak correlation, 0.40 to 0.69 moderate correlation, 0.70 to 0.89 strong correlation, and 0.90 to 1.00 as a very strong correlation.^[16]^

To determine whether the studies included in the meta-analysis have publication bias, primarily the funnel plot was used, and then the Begg and Mazumdar rank correlation statistics were calculated. The Forest plot is shown with 95% CI for the visualization of the marker size according to the study difficulty.

In meta-analysis studies, the choice between a fixed-effect model and a random-effects model is based on investigating the heterogeneity of the studies. For this purpose, the Cochrane *Q* statistic, a chi-square heterogeneity test with (*k −* 1) degrees of freedom, was used to assess heterogeneity.^[17]^ Heterogeneity was evaluated using the *I*^2^ statistic. Depending on the degree of heterogeneity, significance was assessed using both the fixed-effect and random-effects models. An *I*^2^ value of 25% is considered low heterogeneity, 50% is considered moderate heterogeneity, and 75% is considered high heterogeneity. Regarding publication bias analysis, it was concluded that there was no publication bias as the *P* values of the articles were greater than .05.

## 3. Results

As a result of the search made in the specified databases, a total of 1864 articles were reached. Among the articles reached as a result of the search, 185 articles were reached after the titles and abstracts of the 704 articles obtained after removing the duplicates were examined in accordance with the exclusion criteria. Then, from the articles whose full texts were examined in detail, 21 articles were found following our PICO question. After detailed analysis, 5 articles included in this review that had sufficient statistical data to be included in the meta-analysis were found. Scanning was performed according to PRISMA guidelines and outlined in accordance with the PRISMA flowchart (Fig. 1). The main characteristics of the 5 studies included in the meta-analysis are summarized in Table 1.

### 3.1. Analysis of effect sizes, bias analysis, and distribution models of the articles included in the meta-analysis

#### 3.1.1. Analysis of the effect sizes of the articles.

When the effect sizes of the studies are evaluated according to the anatomical regions (Table 2):

**Table 2:** Contingency coefficient values of the studies included in the meta-analysis according to anatomical regions, and bias and heterogeneity evaluations of BIS: 60–65 and BIS: 65–75 subgroups.

	n	BIS	VELUM*	ORAPHARYNX*	TONGUE*	EPIGLOT*
Sang Min Park 2017	85	60–65	0.213	0.1843	0.259	0.745
Yan Wang 2018	85	60–65	0.64	0.583	0.378	0.452
Donghwi Park 2020	26	65–75	0.61	0.5181	0.192	0.713
Bora Bilal 2019	47	65–75	0.562	0.6775	0.561	0.487
Tae-Bin Won 2020	63	70–80	0.595	0.4446	0.295	0.713
Bias						
Kendall’s Tau/*P*			0.105/.79†	0.316/.44†	0.105/.8†	0.104/.79†
Heterogeneity						
*Q*/*P*/*I*^2^ (%)			1.595/.006/72.59‡	15.11/.004/73.53‡	4.99/.29/19.76§	13.11/.01/69.50‡
BIS: 60–65‖						
*Q*/*P*/*I*^2^ (%)			12.0382/.0005/91.69‡	94.687/.0021/89.44‡	0.7219/.39/0.00§	9.2277/.002/89.16‡
BIS: 65–75¶						
*Q*/*P*/*I*^2^ (%)			0.0809/.78/0.00§	0.9496/.33/.00§	2.9226/.09/65.78§	1.97/.16/49.24§

BIS = bispectral index.

* Contingency coefficient.

† No significant bias.

‡ A random-effects model was used due to.

§ A fixed-effects model was used due to homogeneity.

‖ Park et al and Wang et al

¶ Park et al and Bilal et al

##### 3.1.1.1. In the Velum region

Wang et al, Park et al, Bilal et al, and Won et al found a high association in terms of effect sizes as CC>0.5 and 95% CI (CC: 0.64, CC: 0.61, CC: 0.562, and CC: 0.595 respectively). In contrast, Park et al the effect size for the velum region of their study was determined as CC: 0.213 and 95% CI low association.

##### 3.1.1.2. In the Oropharynx region

The studies of Wang et al, Park et al, and Bilal et al found a high association as CC>0.5 and 95% CI (CC: 0.583, CC: 0.518, CC: 0.67 respectively). Won et al the effect size for the oropharynx region of their study was determined as CC: 0.446 and 95% CI as a moderate association. Park et al the effect size for the oropharynx region of their study was determined as CC: 0.184 and 95% CI low association.

##### 3.1.1.3. In the Tongue region

Bilal et al found a high association in their study because the effect size was CC: 0.561 and 95% CI. Wang et al the effect size of their study was determined as CC: 0.378 and 95% CI moderate association. In contrast, Park et al, Park et al, and Won et al, the effect sizes for the tongue region of their study were determined as a low association (CC: 0.259, CC: 0.192, CC: 0.295 respectively and 95% CI).

##### 3.1.1.4. In the Epiglottis

Park et al, Park et al, and Won et al studies found a high association as CC>0.5 and 95% CI (CC: 0.745, CC: 0.713, and CC: 0.713 respectively). Wang et al and Bilal et al the effect sizes of their studies were found as a moderate association (CC: 0.452 and CC: 0.487 respectively and 95% CI).

#### 3.1.2. Analysis of bias analysis and distribution models.

Since the *P* values of the articles were found to be greater than .05 in the publication bias analysis, it was determined that there was no publication bias (Table 2).

When the 5 studies in the current study are analyzed according to their distribution; the random effect as it shows the heterogeneous distribution in the velum (*P* = .006, *I*^2^ = 72.5%), oropharynx (*P* = .004, *I*^2^ = 73.53%), and epiglottis (*P* = .01, *I*^2^ = 69.5%) regions model is selected. However, the fixed-effect model was chosen because it was homogeneously distributed in the tongue root (*P* = .29, *I*^2^ = 19.76%).

### 3.2. Analysis of the included articles in terms of the primary outcome

#### 3.2.1. Analysis of studies according to obstruction areas.

**Figure 2.**
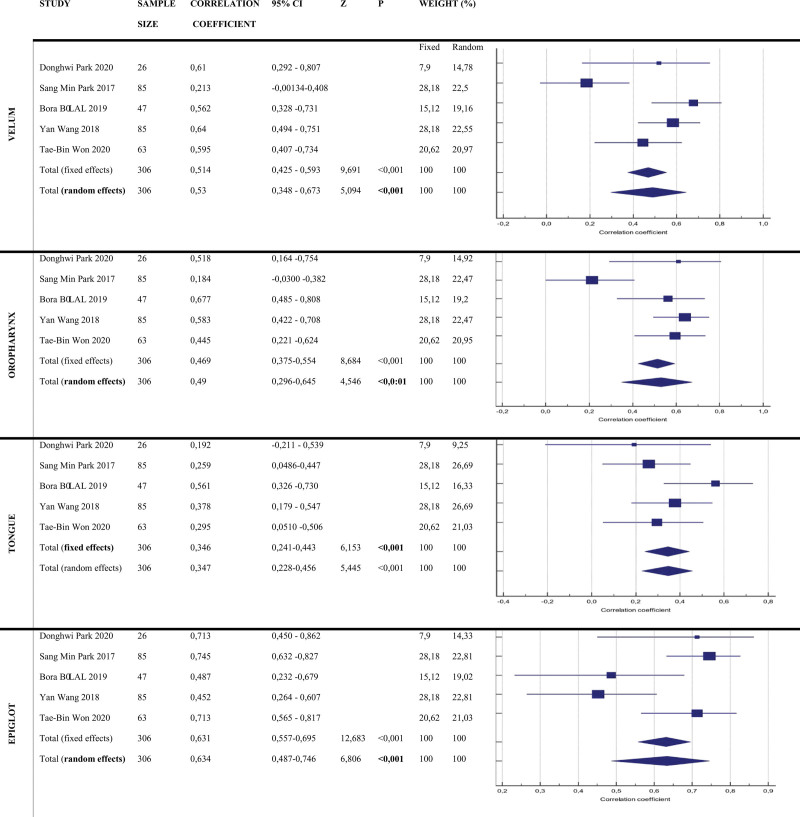
Correlation coefficient values of 5 studies and forest plots.

When evaluating collapsed anatomical areas according to the VOTE score (Fig. 2):

The correlation Coefficient value of the articles examined in the research in the **Velum** region was determined as 0.53 and 95% CI: 0.348 to 0.673. The collapsibility in the Velum region showed a moderate association with the BIS sedation level, as indicated by a correlation coefficient of 0.53. Since the distribution in the velum is heterogeneous, *P* < .0001 was found to be statistically significant according to the random effect model.

The correlation coefficient values of the articles examined in the study in the **Oropharynx** region were found to be 0.49 and 95% CI: 0.296 to 0.645. The collapsibility in the oropharynx region showed a moderate association with the BIS sedation level, as indicated by a correlation coefficient of 0.49. Since the distribution in the velum is heterogeneous, *P* < .0001 was found to be statistically significant according to the random effect model.

The correlation coefficient values of the articles examined in the study in the **Tongue** region were found to be 0.346 and 95% CI: 0.241 to 0.443. The collapsibility in the tongue region showed a weak association with the BIS sedation level, as indicated by a correlation coefficient of 0.346. Since the distribution in the tongue root is homogeneous, *P* < .0001 was found to be statistically significant according to the fixed effect model.

The correlation coefficient values of the articles examined in the study in the **Epiglottis** region were found to be 0.639 and 95% CI: 0.487 to 0.746. The collapsibility in the epiglottis region showed a moderate association with the BIS sedation level, as indicated by a correlation coefficient of 0.639. Since the distribution in the epiglottis region is heterogeneous, *P* < .0001 was found to be statistically significant according to the random effect model.

In the presented meta-analysis, a statistically significant correlation was found between the BIS sedation level and the areas of obstruction in the VOTE score. The strongest association is at the epiglottis level, followed by the velum, oropharynx, and tongue root, respectively (CC: 0.639, CC: 0.53, CC: 0.49, CC: 0.346 and *P* < .001).

### 3.3. Analysis of the included articles in terms of the secondary outcome

#### 3.3.1. Subgroup analysis according to BIS sedation levels.

##### 3.3.1.1. Analysis of publications with BIS sedation levels between 60 and 65 (Park et al and Wang et al)

When the distribution patterns of both studies were examined, the velum (*P* = .0005, *I*^2^ = 91.69%), oropharynx (*P* = .0021, *I*^2^ = 89.44%), and epiglottis (*P* = .002, *I*^2^ = 89.16%) showed heterogeneous distribution and random effect model was applied. However, since there was a homogeneous distribution in the tongue root (*P* = .39, *I*^2^ = 0.00%), the fixed effect model was applied (Table 2).

**Figure 3.**
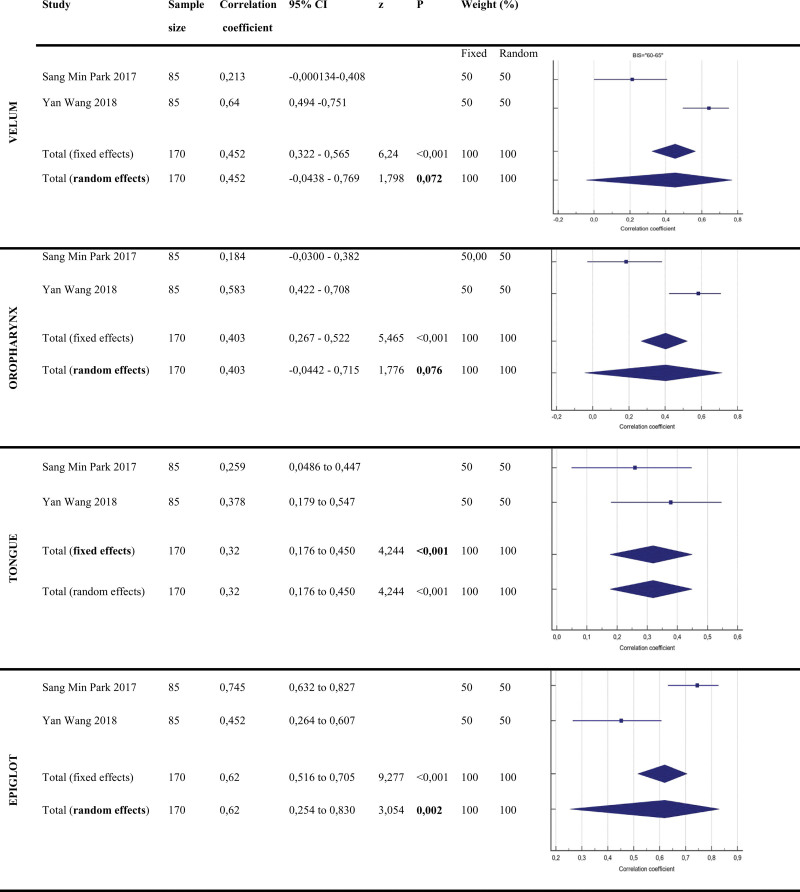
Correlation coefficient values and forest plots of BIS: 60–65 subgroup. BIS = bi-spectral index.

When evaluating the collapsed anatomical areas according to the VOTE score (Fig. 3):

In the **Velum** region, the Correlation Coefficient values of the articles examined in the study were 0.452 and 95% CI: −0.00438 and −0.769. Since the distribution in the velum is heterogeneous, it was not statistically significant according to the random effect model (*P* = .072).

The correlation coefficient values of the articles examined in the study in the **Oropharynx** region were found to be 0.403 and 95% CI: −0.0442 to −0.715. Since the distribution in the oropharynx region is heterogeneous, it was not statistically significant according to the random effect model (*P* = .076).

The correlation coefficient values of the articles examined in the study in the **Tongue** region were found to be 0.32 and 95% CI: 0.176 to 0.450. Since the distribution in the tongue root is homogeneous, *P* < .0001 was found to be statistically significant according to the fixed effect model.

The correlation coefficient values of the articles examined in the study in the **Epiglottis** region were found to be 0.62 and 95% CI: 0.254 to 0.830. Since the distribution in the epiglottis region is heterogeneous, *P* < .0002 was found to be statistically significant according to the random effect model.

##### 3.3.1.2. Analysis of publications (Park et al and Bilal et al) with BIS sedation levels between 65 and 75

When the distribution patterns were examined in both studies, the velum (*P* = .78, *I*^2^ = 0.00%), the oropharynx (*P* = .33, *I*^2^ = 0.00%), the tongue root (*P* = .09, *I*^2^ = 65.78%), and epiglottis (*P* = .16, *I*^2^ = 49.24%) were found to be homogeneously distributed, and fixed-effect model was applied (Table 2).

**Figure 4.**
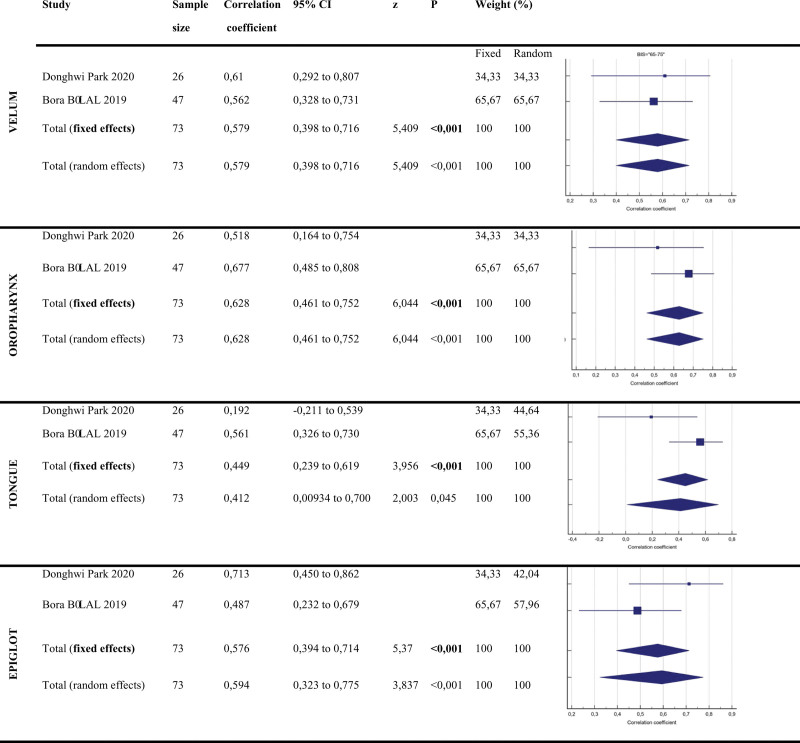
Correlation coefficient values and forest plots of BIS: 65–75 subgroup. BIS = bi-spectral index.

When evaluating the collapsed anatomical areas according to the VOTE score (Fig. 4):

In the **Velum** region, the correlation coefficient values of the articles examined in the study were 0.579 and 95% CI: 0.398 and 0.716. Since the distribution in the velum is homogeneous, it was found statistically significant as *P* < .001 according to the fixed effect model.

The correlation coefficient values of the articles examined in the study in the **Oropharynx** region were 0.628 and 95% CI: 0.461 to 0.752. Since the distribution in the oropharynx region is homogeneous, it was found to be statistically significant at *P* < .001 according to the fixed effect model.

The correlation coefficient values of the articles examined in the study in the **Tongue** region were found to be 0.449 and 95% CI: 0.239 to 0.619. Since the distribution in the tongue root is homogeneous, *P* < .0001 was found to be statistically significant according to the fixed effect model.

The correlation coefficient value of the articles examined in the study in the epiglottis region was determined as 0.576 and 95% CI. Since the distribution in the **Epiglottis** region is homogeneous, *P* < .0001 was found to be statistically significant according to the fixed effect model.

## 4. Discussion

In the presented meta-analysis, A statistically significant correlation was found between the BIS sedation level and the areas of obstruction in the VOTE score. The strongest association is at the epiglottis level, followed by the velum, oropharynx, and tongue root, respectively (CC: 0.639, CC: 0.53, CC: 0.49, CC: 0.346 and *P* < .001).

In the subgroup analysis of publications with BIS sedation levels in the range of 60 to 65, when the velum and oropharynx regions were examined, it was not found to be statistically significant according to the random effect model since the distribution was heterogeneous (*P* = .72, *P* = .76 respectively). The distribution in the epiglottis region was heterogeneous, and it was found to be statistically significant according to the random effect model (*P* < .001). The distribution in the tongue was homogeneous, and it was found to be statistically significant according to the fixed effect model (*P* < .001). However, while the obstruction at the base of the tongue and the sedation level between BIS: 60 to 65 were found to be a weak correlation (CC: 0.32), a moderate correlation was found with the obstruction at the epiglottis level (CC: 0.62).

When the publications in which the BIS sedation level is in the range of 65 to 75 are examined according to the areas of obstruction; at this sedation level, the distribution in 4 anatomical regions was homogeneous and statistically significant according to the fixed effect model (*P* < .001). Although the relationship between the 4 anatomical regions that developed obstruction and BIS: 65 to 75 sedation level was determined as moderate, the highest numerical value was in the oropharynx (CC: 0.628) region. Oropharynx; It was followed by the velum, epiglottis, and root of the tongue (CC: 0.579, CC: 0.576, and CC: 0.449 respectively).

The soft palate, oropharynx, and hypopharynx, which form the upper airway, have different critical closing pressures.^[13]^ Therefore, while some areas collapse at higher pressures, some areas collapse at lower pressures.^[18]^ Anesthetic and sedative agents are effective in the collapse of anatomical regions in OSA patients.^[8]^ Obstruction levels vary depending on the depth of sedation.^[19]^ However, it is not clear to what extent sedation affects collapsibility.^[19]^

Sedation applied during DISE application may change the dynamics of the upper airway during apnea attacks and cause false-positive upper airway collapse due to excessive muscle relaxation.^[20]^ It is necessary to measure the depth of sedation during DISE to simulate sleep closest to natural sleep and to understand muscle relaxation levels.^[9]^ However, there is no standard protocol for the evaluation of collapse or sedation application and monitoring methods in DISE.^[21]^In our meta-analysis, the level of sedation was found to be highly correlated with epiglottic collapse. In addition, in subgroup analyzes, the epiglottic collapse was moderately associated with BIS sedation levels being both in the range of 60 to 65 and in the range of 65 to 75. This can be explained by the fact that obstruction in the epiglottis region is secondary to obstruction in other anatomical regions.^[22]^ In fact, although epiglottic collapse is observed in 12% of all OSA patients on awake patient examination, many studies have shown that this rate is higher than expected in patients who underwent DISE.^[23]^ Due to the multifactorial and multisegmental nature of the collapse process in OSA patients, the nature of obstruction sites can change overnight.^[24]^ However, the higher prevalence of epiglottic collapse can be attributed to the presence of multiple segmental obstructions and the high sensitivity of DISE in diagnosis. DISE has a high ability to detect multi-level obstruction sites even in mildly symptomatic patients (AHI < 5).^[19]^

However, this meta-analysis was based on the VOTE Score and the individual contributions of anatomical regions were investigated. The VOTE classification, developed by Keziran et al based on approximately 8000 experiences, provides a framework for characterizing various structures that contribute to upper airway narrowing and/or obstruction.^[14]^ These structures include the soft palate (velum), oropharyngeal lateral walls, and tongue base, each of which can individually contribute to the obstruction. The VOTE score determines both the presence and the degree of collapse in the identified anatomical regions.

We found that obstruction at the base of the tongue was significant at both sedation levels, but the rate of association increased when the sedation level decreased. In addition, we found that obstruction in the velum and oropharynx region was not statistically significant in the BIS: 60 to 65 sedation range but was significant at a superficial sedation level (BIS: 65–75). At the level of superficial sedation, all 3 sites were moderately associated. Therefore, this level of sedation may identify more obstruction sites. Similar to our research, Viana Ada et al,^[25]^ BIS values in the 53 to 70 range during DISE application were found to be the most affected obstruction area of palatal collapse. Ravesloot and de Vries,^[26]^ on the other hand, found the regions where the obstruction was observed the most, in order of frequency, as palatal collapse (83%), the base of the tongue (56%), epiglottis (38%), and oropharyngeal collapse (7%). Vroegop et al^[27]^ found palatal collapse (81%), the base of the tongue (46.6%), and oropharyngeal collapse (38.7%) rates. In another study on this subject, the rates of obstruction were found to be approximately 85% to 90% for the soft palatal region, 50% to 60% for the tonsil (or lateral pharyngeal wall), and 30% for the tongue root and epiglottis, respectively, according to their anatomical areas.^[27]^ In a study, it was determined that the soft palatine region was the most common occlusion area and that half of the patients had collapsed at the base of the tongue.^[23]^ Although the rates of results differ between studies, during DISE application, velopharynx/retropalatal and tongue/retrolingual collapse is seen in most of the OSA patient group (80–90%), and more superficial sedation allows us to catch up with this patient group.^[27]^

In this meta-analysis study, we conducted a literature review based on the PICO question. The aim was to examine the relationship between the severity of collapse in anatomical regions, as determined by the VOTE score, and BIS sedation levels. The meta-analysis included patients diagnosed with OSA (AHI > 5) according to the American Academy of Sleep Medicine definition.^[28]^ When examining the patient population in this study, it was found that they had similar AHI values and were categorized as having moderate to severe OSA (AHI > 15) and severe OSA (AHI > 30). Ravesloot and de Vries^[26]^ found a correlation between high AHI values and high collapsibility. However, given the limited number of studies on this topic, our research focused on the patient population diagnosed with OUA as defined by AASM. Additionally, there is a relationship between BMI and collapsibility.^[29]^ The patient population included in the presented meta-analysis had similar BMI values ranging from 25 to 30, classified as overweight according to the World Health Organization definition.^[30]^ Although it is known that the upper airway muscles relax mainly during the rapid eye movement period of sleep in OSA patients, the views are not clear as to what range the BIS sedation level is in the REM period.^[31]^ In the literature, studies show the stages of sleep with different results. Nieuwenhuijs et al^[24]^ classified sleep periods according to BIS levels as evoked conditions (98–94), stage 2 sleep (65–44), stage 3 sleep (49–35), and REM sleep (51–44). they detected. Sleigh et al^[32]^ determined light sleep (75–90) for slow-wave sleep (20–70) and REM period (75–92). Dahaba et al^[33]^ suggested that for stage 2 sleep (70–80), stage 3 (50–60), stage 4 (40–50), and REM sleep (60–70). Considering that the half-maximum effect of the collapsibility range for all obstruction patterns and anatomical levels varies between 65 and 80, the BIS: 65 to 75 range can catch the collapse of the anatomical regions included in the VOTE score, as we found in our study.^[31]^

In addition, another condition affecting the collapse patterns may be different doses of different anesthetics/sedatives, even if the BIS is in the same sedation range. Three studies included in the meta-analysis were conducted with midazolam infusion, and 2 studies were conducted with propofol infusion and without using a target-controlled infusion device. Midazolam is a short-acting benzodiazepine. Since midazolam has a muscle relaxation effect, it has been determined that it relaxes more pharyngeal muscles than propofol.^[34,35]^ Propofol is a short-acting hypnotic/amnestic agent. Bolus use during DISE can potentially result in excessive muscle relaxation and respiratory depression.^[31,36]^ In the presented analysis Wang et al (BIS: 60–65) and Bilal et al (65–75) in their study, none of the anatomical regions in the VOTE score were found to be associated with a low level of collapse. The common feature of both studies is that they are performed with propofol infusion without using a TCI device in sedation. This makes us think that the properties of drugs applied other than sedation levels may also be adequate for the degree of obstruction.

Again, the common feature of DISE applications in the publications included in this study is that they are applied while the patients are in the supine position. Collapse patterns may vary in the lateral position. In their study, Lee et al^[37]^ found that the obstruction in the base of the tongue and larynx improved when turned from the supine position to the lateral position. They found that the obstructions seen in the lateral position are usually the obstruction originating from the oropharyngeal lateral wall. All obstruction patterns shown in our analysis are valid for patients in the supine position.

## 5. Limitations

The present study has several limitations. First, although there are many studies in the literature for DISE application, there are not many studies examining the degree or location of upper airway narrowing according to the depth of sedation during DISE. Most studies do not meet the inclusion criteria because of the lack of a standard protocol for DISE, depth of anesthesia, patient position, differences in classification systems (such as two-level versus four-level), the definition of obstruction, and fundamental differences in the classification of outcomes. Therefore, the number of studies included in the presented meta-analysis was limited. Second, the relatively small sample size in the studies and the highly selected group make it difficult to generalize the results to all OSA patients. The third limitation of our study is that although the patient populations in the 5 studies had similar AHI values, they were not exactly the same, such as the moderate and severe groups. However, based on the authors’ knowledge of the literature, no studies were found that specifically examined patient groups with identical AHI values. Therefore, we believe that further research is needed in this regard. Finally, some of the results of the included studies were heterogeneous. In order to reduce this heterogeneity as much as possible, it was tried to compensate by choosing the proposed random-effects model.

## 6. Conclusions

In our study, it was found that BIS sedation levels during DISE application in OSA patients were associated with obstruction of the anatomical regions of the upper airway. The strongest association was found at the epiglottis level, followed by the velum, oropharynx, and tongue root, respectively. According to the findings of the meta-analysis, during superficial sedation range (BIS: 65–75), the collapse of anatomical regions represented by the VOTE score can be identified. However, although an association was observed between collapsibility and BIS sedation levels during DISE application, we believe it is not possible to recommend a specific BIS level for DISE. This is because the relationship between BIS levels and sedation achieved through natural sleep or anesthetic drugs has not been fully elucidated. Therefore, further research is needed to better understand the correlation between BIS sedation values and sleep stages.

## Acknowledgements

The statistical consultancy of the presented meta-analysis was provided by MedicReS Good Biostatistical Consultancy Services. We would also like to thank Professor Arzu Kanik for her valuable contributions to the statistics department.

## Author contributions

**Conceptualization:** Özlem Öner, Mustafa Cenk Ecevit, Ali Necati Gökmen.

**Data curation:** Özlem Öner, Mustafa Cenk Ecevit, Ali Necati Gökmen.

**Formal analysis:** Özlem Öner, Mustafa Cenk Ecevit, Ali Necati Gökmen.

**Investigation:** Özlem Öner.

**Methodology:** Özlem Öner, Mustafa Cenk Ecevit, Ali Necati Gökmen.

**Project administration:** Mustafa Cenk Ecevit.

**Supervision:** Ali Necati Gökmen.

**Validation:** Özlem Öner, Mustafa Cenk Ecevit.

**Writing – original draft:** Özlem Öner, Mustafa Cenk Ecevit, Ali Necati Gökmen.

**Writing – review & editing:** Özlem Öner, Mustafa Cenk Ecevit, Ali Necati Gökmen.
